# The Role of Personality in Explaining Burnout, Work Addiction, and Stress-Related Growth in Prehospital Emergency Personnel

**DOI:** 10.3390/healthcare13020193

**Published:** 2025-01-19

**Authors:** Mihai Alexandru Butoi, Gabriela Vancu, Radiana-Carmen Marcu, Andrei Hermenean, Monica Puticiu, Luciana Teodora Rotaru

**Affiliations:** 1 Emergency Medicine and First Aid Department, Faculty of Medicine, University of Medicine and Pharmacy, 200349 Craiova, Romania; mihai.butoi@rmu.smurd.ro (M.A.B.); luciana.rotaru@umfcv.ro (L.T.R.); 2 Department of Psychology, Faculty of Educational Sciences, Psychology and Social Assistance, University Aurel Vlaicu Arad, Elena Drăgoi Street, No. 2, 310032 Arad, Romania; 3 Department of Psychology, Faculty of Social Sciences, Humanities and Physical Education and Sports, Vasile Goldis Western University of Arad, 310325 Arad, Romania; radi.marcu@yahoo.com; 4 Depression Ward 8, General Psychiatric Department, Aalborg University Hospital South, 9000 Aalborg, Denmark; andrei.hermenean@gmail.com; 5 Department of Emergency, Faculty of Medicine, Vasile Goldis Western University of Arad, 310325 Arad, Romania

**Keywords:** burnout, work addiction, stress-related growth, personality factors, prehospital emergency personnel

## Abstract

**Background/Objectives:** This is a cross-sectional study designed to explore the contribution of personality factors (the Alternative Five Factor Model) and lower order characteristics (responsive distress and self-discipline) to burnout, work addiction, and stress-related growth among Romanian prehospital emergency healthcare workers. **Methods:** A total of 266 prehospital professionals (41 physicians, 74 nurses, and 151 paramedics) participated in the study out of the 728 invited (36.5% response rate). The age of participants ranged between 20 and 57 years and 67.3% were men. The participants completed the Oldenburg Burnout Inventory, the Dutch Work Addiction Scale (the short version), the Stress-Related Growth Scale, the Zuckerman–Kuhlman Personality Inventory, the Responsive Distress Scale, and the Self-Discipline Scale. **Results:** The results show that burnout was predicted by age, gender, impulsive sensation seeking, responsive distress, and self-discipline; work addiction was predicted by aggression–hostility, sociability, and responsive distress, while stress-related growth was predicted by age, activity, and self-discipline. Additionally, we found that emergency healthcare workers displayed lower impulsive sensation seeking, neuroticism–anxiety, and aggression–hostility, but displayed higher levels of activity and sociability in comparison with the general Romanian population, although these differences must be interpreted with caution as the general Romanian population tend to be significantly younger. **Conclusions:** This study provides new insights into the role of personality traits as risk factors for burnout and work addiction, and as protective factors for stress-related growth in prehospital emergency personnel. It is also among the few studies in this field to employ the Alternative Five Factor Model of personality.

## 1. Introduction

Managing burnout and stress is essential for prehospital emergency professionals due to their challenging workloads, frequent exposure to high-pressure situations, constant encounters with death and trauma and risks of violence, and the need to make critical, life-saving decisions under tight time constraints. These factors require constant readiness to intervene, impacting not only the physical and mental health of healthcare providers but also the quality of care delivered to patients [1]. Furthermore, burnout carries significant professional consequences, including job dissatisfaction, increased errors, absenteeism, staff turnover, and inefficient resource utilization [2,3].

The concept of burnout was first used by Herbert Freudenberg in relation to caring professionals who experienced high levels of stress and chronic fatigue [4] and was later developed by Maslach who defined it as a tridimensional syndrome characterized by emotional exhaustion, depersonalization, and low personal accomplishment [5].

Currently, two scientifically validated models of burnout are widely recognized: the Maslhach model and the job demands–resources model (the JD-R model) [5]. The Maslach model reflects a three-dimensional syndrome which refers to emotional exhaustion, depersonalization, and low personal achievement and was developed mainly within human services-oriented professions [5]. The JD-R model states that burnout has two dimensions: exhaustion, related to job demands; and disengagement, related to low job resources. When job demands are high and job resources are low, there is a great risk of burnout [5]. Within both models, exhaustion refers to intense physical, emotional, cognitive, or social strain due to work stressors, while disengagement refers to the need to distance oneself from work due to the experience of negative attitudes regarding the work content, the work environment, or just work in general [5].

In the present research, we opted to use the JD-R model, based on two reasons: (1) the JD-R model was developed as a general burnout model and is not related only to human services professions; and (2) several authors argued that the personal accomplishment dimension of the Maslach model is more likely an individual outcome of burnout and not a component of it, as it correlates weakly with emotional exhaustion and depersonalization [5]. The levels of burnout for the JD-R model can be assessed with the Oldenburg Burnout Inventory (OLBI) [5,6].

Burnout rates among emergency personnel over the past two decades have shown significant variation, ranging from moderate to high levels [6,7,8,9,10,11,12,13,14]. A recent review from 2023 reported an overall burnout rate of 43%, with 39% and 43% for exhaustion and depersonalization, respectively [10]. A nationwide Romanian study [15] found moderate to high levels of burnout for 4693 EM health workers (representing 29.94% of the national emergency medical personnel). Additionally, a more recent study conducted in Galați County (Romania) during the COVID-19 pandemic revealed exhaustion rates of 30.6% for nurses and 45.8% for physicians [16].

Burnout across different populations is influenced by both work-related factors (working shifts/hours, work environment, field experience, and non-clinical duties) and non-work-related (individual traits, age, gender, and lifestyle factors) [8,13]. A study involving 263 Romanian emergency professionals found that professional experience was significantly correlated with burnout levels, while factors such as gender, age, and marital status showed no significant association [17].

To date, research on burnout has been focused predominantly on work-related factors, with less emphasis on personality variables, suggesting that personality traits are less relevant. This might be due to the fact that this construct was initially defined and developed as a stress-related variable within stressful professions, and thus, is profoundly connected with the working environment and not the individual [5,18].

However, Bianchi [18], in a study involving 1759 individuals, discovered that neuroticism explained 53.46% of the variance in burnout—exceeding the influence of any tested work-related variable. This finding suggests that the importance of personality traits in the literature on burnout may be underestimated. Personality traits have been shown not only to correlate with burnout levels but also to moderate the relationship between work-related factors and burnout [19,20,21]. A meta-analysis of 114 samples found that personality traits (except openness) were strongly associated with burnout, with five personality factors explaining 29%, 26%, and 23% of the variance in exhaustion, depersonalization, and personal accomplishment, respectively [22]. Similarly, a 2023 review of 83 studies, showed that high neuroticism and low agreeableness, conscientiousness, extraversion, and openness are associated with a higher risk of burnout [23].

For emergency medical (EM) professionals, neuroticism appears to be the main risk factor for burnout, yet other factors contribute nonetheless [1]. A systematic review of prospective studies on risk and protective factors for EM personnel [24] found that neuroticism was significantly associated with future burnout and general poor health. For paramedics and ambulance personnel, neuroticism was significantly associated with emotional exhaustion, and psychological distress [25]. Additionally, a review of 27 studies involving 9721 paramedics highlighted a distinct personality profile characterized by high conscientiousness, sensation seeking, resiliency, and empathy, but low extroversion, neuroticism, and agreeableness [26].

Another consequence of highly stressful professions and working environments is how individuals engage in work activities and how work affects their lives. This idea is not new as it was first tackled by Oates in the early 1970s under the name of workaholism [27]. Researchers considered workaholism to be a lower-order personality trait, defined as a stable pattern of behavior characterized by high energy and high work involvement and strongly correlated with obsessiveness, compulsiveness, and conscientiousness, while work addiction was considered to be the dysfunctional/pathological expression of workaholism [27,28].

Work addiction is characterized by excessive involvement in work that surpasses job requirements or the pursuit of job satisfaction, leading to physical and mental health issues [27]. It is influenced by a combination of individual and work-related factors, which interact closely rather than operating independently. This interconnectedness can have adverse effects not only on psychosocial well-being but also on physical health [27]. Some researchers consider work addiction as the dysfunctional expression of a lower-order personality trait called workaholism, where the latter is defined as a stable pattern of behavior characterized by high energy and high work involvement, strongly correlated with obsessiveness, compulsiveness, and conscientiousness [28].

As a member of the behavioral addiction family, work addiction is described by six core components: salience (work is regarded as the most important activity in one’s life, controlling one’s thoughts, feelings, and actions), mood modification (work becomes a coping strategy), high tolerance (the individual needs increased amounts of work to obtain the same psychological outcome), withdrawal signs (when unable to work, one experiences negative feelings, restlessness, and physical symptoms), conflict with other activities (work interferes with other activities), and relapse (the tendency to return to dysfunctional work patterns after holidays/non-working periods) [29].

Work addiction rates among healthcare professionals vary widely, ranging from low to moderate levels [30,31]. In a previous study [32], we identified that 35% of 266 prehospital emergency personnel (physicians, nurses, and paramedics) exhibited signs of work addiction. Similarly, in a study of 219 Spanish emergency and critical care nurses, 28.3% showed high work addiction scores [33].

Research indicates that high levels of neuroticism, extraversion, and conscientiousness are significant risk factors for work addiction [29]. Neuroticism is linked with work addiction, but the relation is far from straightforward, being mediated by other individual and work-related factors [34]. Other studies have found associations between work addiction and neuroticism and extraversion, but not with psychoticism [35], and also associations between work addiction and spitefulness from the dark personality model [36]. Career-related variables, including career insecurity, barriers, commitment, career goals, and perceived organizational support, also play a role in influencing work addiction [37].

Stress-related growth refers to the positive outcomes one undergoes when exposed to stressful events [38]. Stress-related growth has been observed even in extremely stressful situations like the death of a beloved person, severe illness, divorce, or accidents [39], and even during the recent COVID-19 pandemic [40,41]. Stress-related growth operates on three distinct levels: improved social relations, increased trust in personal resources, and enhanced coping abilities [39,42].

Many factors influence stress-related growth, such as gender, age, ethnicity, coping strategies, social and instrumental support, the duration and severity of stress, and personality characteristics [43,44]. Previous studies show strong correlations between posttraumatic growth and the Big Five factors, yet the relation is mediated by levels of coping [45]. Park et al. developed and validated the Stress-Related Growth Scale (SRGS) as a tool to assess the construct and found that it is significantly predicted by religiousness/spirituality, social support satisfaction, stress intensity, positive reinterpretation and acceptance as coping strategies, and recent positive life events [39]. In a prior study [32] we found that 61.2% of 266 Romanian emergency personnel reported high levels of stress-related growth.

The human personality can be described in several ways, one of the most widely used approaches being the trait model. Among these, the Big Five model is by far the most used in research, though other scientifically validated trait models exist. In our study we use the Alternative Five Factor Model (AFFM) developed by Marvin Zuckerman [46] because it refers to more basic personality factors (temperament-like dimensions) with a strong biological–evolutionary basis [47], and we intended to understand how stress-related variables connect with the more basic human traits. This model describes personality across five broad domains: sociability, neuroticism–anxiety, impulsive sensation seeking, activity, and aggression–hostility [46].

We also assessed two lower-order personality traits: responsive distress and self-discipline. Responsive distress refers to the tendency to experience negative emotions when faced with others’ distress, closely related to empathy [48]. Self-discipline derives from the California Psychological Inventory and refers to one’s ability to self-control and to direct his/her behavior towards rules and procedures compliancy, emphasizing actions over emotions [49].

As shown above, several studies have investigated burnout levels of emergency health workers, but fewer have focused on work addiction and stress-related growth in this professional field. Similarly, the relationship between personality and burnout has been investigated far more than the relationship between personality, work addiction, and stress-related growth among emergency professionals. We believe our study will bring valuable new data to this field by highlighting personality traits with stronger predicting value for burnout, work addiction, and stress-related growth, as well as helping design training programs focused on improving self-discipline and enhancing activity levels.

This study aims to explore how personality traits contribute to burnout, work addiction, and stress-related growth among prehospital emergency healthcare workers who operate in a highly demanding professional setting. Furthermore, it seeks to compare the personality profiles of emergency personnel to the general Romanian population.

Our main hypothesis is that the basic personality dimensions have predictive power in explaining burnout, work addiction, and stress-related growth among emergency health workers. The second hypothesis argues that prehospital emergency personnel tend to show significantly different personality profiles than the general population.

## 2. Materials and Methods

### 2.1. Participants and Procedure

Our study involved 266 prehospital emergency professionals (physicians, nurses, and paramedics), all presently working within the Inspectorate for Emergency Situations (ISU). A survey was distributed to 728 emergency personnel (170 physicians, 400 nurses, and 158 paramedics) across 5 of the 42 national ISU departments (counties) between December 2023 and February 2024. A total of 266 individuals (41 physicians, 74 nurses, and 151 paramedics) completed the survey in full, resulting in a response rate of 36.5%.

The online survey had two sections: the first section focused on demographic and professional characteristics (age, gender, field experience, type of emergency personnel, other personal, and professional variables), while the second included six validated assessment tools (the Oldenburg Burnout Inventory—OLBI, the Dutch Work Addiction Scale, short version—DUWAS-10, the Stress-Related Growth Scale—SRGS, the Zuckerman–Kuhlman Personality Questionnaire—ZKPQ, the Responsive Distress Scale—RDS, and the Self-discipline Scale—SDS).

### 2.2. Measures

The Oldenburg Burnout Inventory (OLBI) is a 16-item self-reported scale designed to assess burnout, including disengagement and exhaustion. These can be assessed by 8 items scored on a 4-point scale (1—strongly disagree to 4—strongly agree). Dimensions scores range from 8 to 32, while the total score ranges from 16 to 64, with higher scores indicating higher levels of exhaustion, disengagement, or burnout [49,50]. Reliability coefficients for the present sample were as follows: 0.826 for the total score, 0.444 for disengagement, and 0.817 for exhaustion (Cronbach’s Alpha).

The Dutch Work Addiction Scale-short version (DUWAS-10) is designed to assess work addiction and its two dimensions: working excessively (WE) and working compulsively (WC). The subscales consist of 5 items each, with items scored on a 4-point scale (1—never to 4—always). The dimensions and total scores are obtained by dividing the raw scores per number of items. The threshold for relevant scores is the 75th percentile [51,52]. For our sample, the reliability for DUWAS-10 was good, with Cronbach’s Alpha values of 0.881 for the total score, 0.805 for the working excessively dimension, and 0.693 for the working compulsively dimension.

The Stress-related Growth Scale (SRGS), developed by Park et al. [39], is a 15-item measure designed to assess stress-related growth. Items are scored on a 3-point scale, where 0—disagree, 1—somewhat agree, and 2—strongly agree. Scores can range from 0 to 30. Scores of 28 or higher indicate relevant stress-related growth, with a Cronbach’s Alpha of 0.855.

The Zuckerman–Kuhlman Personality Questionnaire (ZKPQ), Romanian version, has 99 items with dichotomous response (true versus false), scoring on five scales: sociability—17 items, impulsive sensation seeking—19 items, neuroticism–anxiety—19 items, activity—17 items, and aggression–hostility—17 items, and one additional scale, desirability—10 items [53].

The Responsive Distress scale is part of the IPIP Emotional Intelligence 7 components proposed by Barchard [48]. The Romanian version, retrieved from the International Personality Item Pool (IPIP) at https://researchcentral.ro (15 October 2024), has 10 items with the dichotomous responses yes versus no. Total scores can range between 0 and 10, with higher scores showing greater responsive distress. In our sample, Cronbach’s Alhpa for RDS was 0.955.

The Self-Discipline Scale (SDS) was originally developed by Gough as part of the California Personality Inventory [49]. The Romanian version, retrieved from the International Personality Item Pool (IPIP) at https://researchcentral.ro (accessed on 15 October 2024). has 10 items with yes or no responses, with scores ranging between 0 and 10, and with higher scores reflecting better self-discipline. For our sample, the Cronbach’s Alpha for SDS was 0.469.

### 2.3. Statistical Procedures

Data collected from the online form were systematized and analyzed using IBM SPSS Statistic 20 software. The *p* value was set at 0.05. Both descriptive and inferential statistics were applied.

## 3. Results

A total of 266 adults were included in our study (41 physicians—15.4%, 74 nurses—27.8%, and 151 paramedics—56.8%); 67.3% were men, with age ranging between 20 and 57 years, the mean (M) age was 38.71 ± 9.18 years, and the median age was 40 years. Professional experience ranged from 1 to 38 years, the mean value was 12.32 ± 8.04 years, and the median = 13 years. Women were significantly older than men (M_women_ = 42.25 years; M_men_ = 36.99 years; *p* < 0.001), and more experienced (M_women_ = 14.20 years; M_men_ = 11.41 years; *p* = 0.008).

Table 1 presents descriptive indicators for assessed variables. Men scored significantly higher scores for burnout (*p* < 0.001), disengagement (*p* < 0.001), exhaustion (*p* < 0.001), neuroticism–anxiety (*p* < 0.001), activity (*p* = 0.033), responsive distress (*p* < 0.001), and self-discipline (*p* < 0.001). Women scored higher on work addiction (*p* < 0.001), working excessively (*p* < 0.001), working compulsively (*p* = 0.019), stress-related growth (*p* = 0.003), impulsive sensation seeking (*p* = 0.015), and sociability (*p* = 0.006). Differences were tested with the U Mann–Whitney test, as many variables do not follow the normal standard distribution law.

Age positively correlates (rho Spearman) with work addiction (*p* < 0.001), working excessively (*p* < 0.001), working compulsively (*p* = 0.003), stress-related growth (*p* < 0.001), activity (*p* < 0.001), and sociability (*p* < 0.001), and negatively with burnout (*p* < 0.001), disengagement (*p* < 0.001), exhaustion (*p* < 0.001), impulsive sensation seeking (*p* < 0.001), neuroticism–anxiety (*p* < 0.001), aggression–hostility (*p* < 0.001), and responsive distress (*p* < 0.001).

Table 2 shows the correlations between the outcome variables. Burnout and its two components negatively correlate with work addiction and working excessively, but not with working compulsively, except for disengagement. Burnout and exhaustion negatively correlate with stress-related growth. Work addiction and its two components positively correlate with stress-related growth. 

Table 3 shows correlations between personality factors and traits. Strong correlations between the five factors are observed, with positive ones between neuroticism–anxiety and impulsive sensation seeking and aggression–hostility, activity, and sociability, and negative ones between impulsive sensation seeking, neuroticism–anxiety, and aggression–hostility on one hand, and activity and sociability on the other hand. Neuroticism–anxiety and aggression–hostility positively correlate with responsive distress. Aggression–hostility, activity, and responsive distress positively correlate with self-discipline but negatively correlate with sociability and impulsive sensation seeking.

To test the main hypothesis, we performed hierarchical multiple regressions, with burnout, work addiction, and stress-related growth as dependent variables, and three sets of predictors, socio-demographic and professional variables (gender, age, and experience), personality domains (the alternative five factors), and lower order personality traits (responsive distress and self-discipline). The results are synthesized in Table 4. Since the variables did not follow a normal distribution, we tested the residual distribution using a Q-Q plot. The results indicated a normal distribution for burnout, disengagement, exhaustion, and stress-related growth, but not for work addiction, working excessively, and working compulsively.

The variance explained by the models ranges between 7% (working compulsively) and 59.8% (stress-related growth). The relevant predictors for burnout and exhaustion are gender, age, impulsive sensation seeking, and self-discipline, while for disengagement: gender, age, responsive distress, and self-discipline. For work addiction and both its components, predictors are aggression–hostility, sociability, and responsive distress. Stress-related growth is predicted by age, activity, and self-discipline. The results support our hypothesis.

To test the second hypothesis, we compared personality factors scores from our sample with those of the general sample involved in the adaptation process for ZKPQ in the Romanian population [53]. The national sample is significantly younger than our sample (*p* < 0.001). The mean age in the national sample is 26.72 years for men, and 27.59 years for women, compared to 36.99 years for men and 42.25 years for women in our sample. Cohen’s d values range from 0.31 (impulsive sensation seeking, women) to 2.19 (sociability, women). Differences between the national sample and our sample are significant for all five factors, with a moderate to large effect size and for both men and women, supporting our second hypothesis.

## 4. Discussion

The main goal of our study was to better understand the role that personality plays in stress-related issues associated with emergency health workers. Our findings show that personality plays a significant role, but the extent varies. For negative stress-related outcomes (such as burnout and work addiction), personality accounts for a smaller proportion of the variance compared to work-related factors, suggesting that the latter have a greater impact. In contrast, personality plays a more substantial role in the positive outcome of stress-related growth, highlighting its importance in fostering growth through stressful experiences.

Work addiction and its components seem to be the least influenced by personality, with variances below 11%. High aggression–hostility and sociability, and low responsive distress are significant predictors for work addiction and excessive and compulsive work. However, these results should be interpreted with caution, as the residuals do not follow a normal distribution.

Previous studies using five-factor models show mixed results. Griffiths et al. [27] argue that several studies found consistent associations between work addiction and neuroticism, but not for the other four factors. In a meta-analysis on work addiction and personality across different populations [29], significant associations were found for extraversion, conscientiousness, and intellect/imagination, but not for neuroticism and agreeableness. For our sample, neuroticism was not a significant predictor, yet aggression–hostility and sociability were. This may be due to the use of another affective trait, responsive distress, which is strongly correlated with neuroticism–anxiety (rho Spearman = 0.274, *p* < 0.001), and served as a significant predictor of work addiction in our analysis. Additionally, neuroticism–anxiety levels in our sample were significantly lower than those observed in the general Romanian population, as supported by our second hypothesis. This suggests that for emergency personnel, neuroticism might not be the driving force for the risk of work addiction.

High aggression–hostility might act as a risk factor for work addiction by driving individuals to engage more in work activities due to their stressful interpersonal relationships, yet it might also be an outcome of addiction, as proposed by Griffiths et al. [27]. The influence of high sociability may be mediated by the work environment. Individuals with high sociability may become more engaged in work activities due to positive relationships with their colleagues, seeking social rewards rather than the rewards associated with work itself. This is strongly supported by our finding that sociability levels in our sample were significantly higher than in the general population (see Table 5).

Burnout and its dimensions are moderately determined by personality, with variances ranging from 26.6% (disengagement) up to 39.2% (burnout). Somville et al. [6] report that previous studies show that personality accounts for up to 60% of the variance in burnout. Our findings indicate that men and younger EM professionals are at a greater risk of burnout. Consistent with earlier studies [54,55], age is negatively associated with burnout; however, some populations have shown differing trends, with a linear relationship for men and a bimodal pattern for women [56]. The higher burnout risk among men in our study could be attributed to the large number of paramedics in the sample, who are predominantly male (98%) and exhibit higher burnout levels compared to nurses and physicians [32].

Among the emergency personnel studied, the personality risk factors for burnout and exhaustion were identified as low impulsive sensation seeking and high self-discipline, while disengagement was associated with low responsive distress and high self-discipline. Individuals with low impulsive sensation seeking behavior are more organized, less spontaneous, and more prone to being worn out by excessive stimuli. In the field of prehospital emergency medicine—characterized by heavy workloads, time pressure, uncertainty, and high-risk situations—these traits can be disadvantageous. This is because the stimulation levels of the environment are generally high but not constant, and planning often fails in unpredictable environments. Impulsive sensation seeking is negatively correlated with conscientiousness, while self-discipline is positively associated with conscientiousness, the latter being linked to burnout in numerous studies across various populations [1,22,23,57,58,59,60]. Additionally, individuals with low responsive distress are less emotionally engaged in their work, making them more susceptible to disengagement from work-related responsibilities.

Stress-related growth is strongly predicted by age, high activity, and high self-discipline. Older, more active, and better-disciplined emergency professionals are more likely to experience positive outcomes from exposure to stressful situations. This may be attributed to the fact that they have had sufficient time and behavioral resources to develop more effective coping strategies, such as problem-solving and seeking social support. These results may be used to improve selection procedures for emergency personnel, but also for training by focusing on improving self-discipline through better work-organization and the stricter use of procedures, and on stimulating them to be more active in and outside of the work environment.

Emergency health workers seem to have a distinct personality profile in comparison with the general population, characterized by lower neuroticism–anxiety, impulsive sensation seeking, and aggression–hostility, and higher activity and sociability. This specific profile could explain their professional choice in the field of medicine, yet it can also be an adaptation to a very stressful activity, or a combination of the two. However, these findings should be interpreted with caution, as our sample is significantly older than the national reference sample.

## 5. Conclusions

Our findings indicate that personality traits, alongside age and gender, play a significant role in stress-related growth and burnout, but less in work addiction, among prehospital emergency healthcare workers. Predictors for stress-related growth were age, activity, and self-discipline. Burnout was predicted by gender, age, impulsive sensations seeking, and self-discipline, while work addiction was predicted by aggression–hostility, sociability, and responsive distress. Additionally, we identified a distinct personality profile for this population, characterized by lower levels of neuroticism, anxiety, aggressiveness, hostility, and sensation seeking, and higher levels of activity and sociability.

This study provides new insights into the role of personality traits as risk factors for burnout and work addiction, and as protective factors for stress-related growth in prehospital emergency personnel. It is also among the few studies in this field to employ the Alternative Five Factor Model of personality. Future research should focus on exploring differences in personality traits among various types of prehospital emergency personnel.

## Figures and Tables

**Table 1:** Descriptive statistics for burnout, work addiction, stress-related growth, and personality factors.

Measure	Mean	Standard Deviation	Median	Minimum	Maximum
Burnout	53.41	9.68	58.0	17	64
Disengagement	26.78	3.97	29.0	9	32
Exhaustion	26.63	6.36	29.0	8	32
Work addiction	2.39	0.90	1.9	1.0	3.7
Working excessively	1.96	1.03	1.6	1.0	3.4
Working compulsively	2.82	0.83	2.2	1.0	4.0
Stress-related growth	26.48	4.80	30.0	13	30
Sociability	13.11	3.39	14.0	2	17
Impulsive sensation seeking	9.26	3.46	8.0	4	18
Neuroticism–anxiety	4.60	3.08	3.0	1	12
Activity	11.62	3.96	13.0	2	16
Aggression–hostility	2.52	2.10	2.0	1	8
Responsive distress	4.40	1.31	5.0	1	8
Self-discipline	9.63	0.77	10.0	5	10

**Table 2:** Correlations between outcome variables.

N = 266		Disengagement	Exhaustion	Work Addiction	Working Excessively	Working Compulsively	Stress-Related Growth
Burnout	rho Spearman	0.843	0.873	−0.165	−0.189	−0.096	−0.126
	*p* (2-tailed)	0.000	0.000	0.007	0.002	0.119	0.040
Disengagement	rho Spearman		0.546	−0.211	−0.221	−0.156	−0.083
	*p* (2-tailed)		0.000	0.001	0.000	0.011	0.177
Exhaustion	rho Spearman			−0.129	−0.164	−0.064	−0.164
	*p* (2-tailed)			0.035	0.007	0.300	0.007
Work addiction	rho Spearman				0.972	0.941	0.319
	*p* (2-tailed)				0.000	0.000	0.000
Working excessively	rho Spearman					0.877	0.284
	*p* (2-tailed)					0.000	0.000
Working compulsively	rho Spearman						0.331
	*p* (2-tailed)						0.000

**Table 3:** Correlations between personality factors.

N = 266		Neuroticism–Anxiety	Aggression–Hostility	Activity	Sociability	Responsive Distress	Self-Discipline
Imp sensation seeking	rho Spearman	0.492	0.407	−0.543	−0.404	−0.083	−0.133
	*p* (2-tailed)	0.000	0.000	0.000	0.000	0.175	0.030
Neuroticism–anxiety	rho Spearman		0.737	−0.420	−0.506	0.274	0.106
	*p* (2-tailed)		0.000	0.000	0.000	0.000	0.085
Aggression–hostility	rho Spearman			−0.482	−0.619	0.239	0.137
	*p* (2-tailed)			0.000	0.000	0.000	0.026
Activity	rho Spearman				0.339	0.114	0.167
	*p* (2-tailed)				0.000	0.063	0.006
Sociability	rho Spearman					−0.198	−0.127
	*p* (2-tailed)					0.001	0.038
Responsive distress	rho Spearman						0.239
	*p* (2-tailed)						0.000

**Table 4:** Results for multiple hierarchical regression.

	Burnout	Disengagement	Exhaustion	Work Addiction	Working Excessively	Working Compulsively	Stress-Related Growth
Model (R^2^ adjusted)	0.392	0.266	0.389	0.090	0.109	0.070	0.598
Gender	t = −6.008	t = −4.155	t = −6.456	t = 1.137	t = 1.919	t = 0.133	t = −0.486
	*p* < 0.001	*p* < 0.001	*p* < 0.001	*p* = 0.257	*p* = 0.056	*p* = 0.894	*p* = 0.627
Age	t = −3.957	t = −3.613	t = −3.652	t = 0.797	t = 0.911	t = 0.619	t = 5.958
	*p* < 0.001	*p* < 0.001	*p* < 0.001	*p* = 0.426	*p* = 0.363	*p* = 0.536	*p* < 0.001
Experience	t = 1.040	t = 0.489	t = 1.276	t = 0.116	t = −0.047	t = 0.309	t = 0.482
	*p* = 0.299	*p* = 0.625	*p* = 0.203	*p* = 0.908	*p* = 0.962	*p* = 0.758	*p* = 0.630
Impulsive sensation seeking	t = −2.271	t = −1.648	t = −2.387	t = −0.721	t = −0.730	t = −0.674	t = −0.525
	*p* = 0.024	*p* = 0.100	*p* = 0.018	*p* = 0.472	*p* = 0.466	*p* = 0.501	*p* = 0.600
Neuroticism–anxiety	t = −1.097	t = −1.122	t = −0.931	t = −0.631	t = −1.005	t = −0.146	t = −1.846
	*p* = 0.274	*p* = 0.263	*p* = 0.353	*p* = 0.529	*p* = 0.316	*p* = 0.884	*p* = 0.066
Aggression–hostility	t = 0.322	t = −0.675	t = 0.961	t = 2.121	t = 2.047	t = 2.107	t = −0.052
	*p* = 0.748	*p* = 0.500	*p* = 0.338	*p* = 0.035	*p* = 0.042	*p* = 0.036	*p* = 0.958
Activity	t = 0.267	t = −0.053	t = 0.450	t = 0.040	t = −0.665	t = 0.892	t = 2.696
	*p* = 0.790	*p* = 0.958	*p* = 0.653	*p* = 0.968	*p* = 0.507	*p* = 0.373	*p* = 0.007
Sociability	t = −0.986	t = −1.410	t = −0.561	t = 2.234	t = 2.008	t = 2.399	t = −0.106
	*p* = 0.325	*p* = 0.160	*p* = 0.575	*p* = 0.026	*p* = 0.046	*p* = 0.017	*p* = 0.915
Responsive distress	t = −0.075	t = −2.075	t = 1.305	t = −2.612	t = −2.369	t = −2.780	t = −0.598
	*p* = 0.940	*p* = 0.039	*p* = 0.193	*p* = 0.010	*p* = 0.019	*p* = 0.006	*p* = 0.550
Self-discipline	t = 4.528	t = 4.341	t = 4.038	t = 0.890	t = 1.056	t = 0.645	t = 2.358
	*p* < 0.001	*p* < 0.001	*p* < 0.001	*p* = 0.374	*p* = 0.292	*p* = 0.520	*p* = 0.019

**Table 5:** Emergency personnel sample versus general population.

Factor	Gender	National Sample			Emergency Sample			Difference		Cohen’s d
		N	M	SD	N	M	SD	t	*p*	
Impulsive sensation seeking	men	305	11.41	3.97	179	9.21	3.67	6.05	<0.001	0.57
	women	520	10.49	4.11	87	9.37	3.01	2.43	0.015	0.31
Neuroticism–anxiety	men	305	6.29	4.21	179	5.03	3.20	3.46	<0.001	0.34
	women	520	8.95	4.32	87	3.71	2.66	10.97	<0.001	1.46
Aggression–hostility	men	305	7.37	3.66	179	2.72	2.16	15.48	<0.001	1.54
	women	520	7.29	3.65	87	2.13	1.93	12.88	<0.001	1.76
Activity	men	305	10.01	3.25	179	11.73	4.23	5.01	<0.001	0.45
	women	520	9.43	3.49	87	11.40	3.36	4.90	<0.001	0.57
Sociability	men	305	8.74	3.99	179	12.64	3.66	10.70	<0.001	1.02
	women	520	7.83	3.87	87	14.98	2.52	14.89	<0.001	2.19

M—mean; SD—standard deviation.

## Data Availability

The data presented in the study are available on request from the corresponding author.

## References

[1] Bahadori M., Ravangard R., Raadabadi M., Hosseini-Shokouh S.M., Behzadnia M.J. (2019). Job Stress and Job Burnout Based on Personality Traits among Emergency Medical Technicians. Trauma Mon..

[2] Donnelly E. (2012). Work-related stress and posttraumatic stress in emergency medical services. Prehospital Emerg. Care.

[3] Stehman C.R., Testo Z., Gershaw R.S., Kellogg A.R. (2019). Burnout, Drop Out, Suicide: Physician Loss in Emergency Medicine. Part I West J. Emerg. Med..

[4] Rajvinder S. (2018). Brief history of burnout. Br. Med. J..

[5] Demerouti E., Bakker A.B., Nachreiner F., Schaufeli W.B. (2001). The Job demands-sources model of burnout. J. Appl. Psychol..

[6] Somville F., Van der Mieren G., De Cauwer H., Van Bogaert P., Franck E. (2022). Burnout, stress and Type D personality amongst hospital/emergency physicians. Int. Arch. Occup. Environ. Health.

[7] Potter C. (2006). To what extent do nurses and physicians working with emergency department experience burnout: A review of the literature. Aust. Emerg. Nurs. J..

[8] Arora M., Asha S., Chinnappa J., Diwan A.D. (2013). Burnout in emergency medicine physicians: Review. Emerg. Med. Australas..

[9] Verougstraete D., Hachimi Idrissi S. (2020). The impact of burn-out on emergency physicians and emergency medicine residents: A systematic review. Acta Clin. Belg..

[10] Alanazy A.R.M., Alruwaili A. (2023). The Global Prevalence and Associated Factors of Burnout among Emergency Department Healthcare Workers and the Impact of the COVID-19 Pandemic: A Systematic Review and Meta-Analysis. Healthcare.

[11] Adriaenssens J., De Gucht V., Maes S. (2015). Determinants and prevalence of burnout in emergency nurses: A systematic review of 25 years of research. Int. J. Nurs. Stud..

[12] Khashaba E.O., El-Sherif M.A.F., Ibrahim A.A.-W., Neatmatallah M.A. (2014). Work-related psychosocial hazards among emergency medical responders (EMRS) in Mansoura city. Indian J. Community Med. Off. Publ. Indian Assoc. Prev. Soc. Med..

[13] Abdo S.A., El-Sallamy R.M., El-Sherbiny A.A., Kabbash I.A. (2016). Burnout among physicians and nursing staff working in the emergency hospital of Tanta University, Egypt. East Mediterr. Health J..

[14] Teixeira C., Ribeiro O., Fonseca A.M., Carvalho A.S. (2013). Burnout in intensive care units—A consideration of the possible prevalence and frequency of new risk factors: A descriptive correlational multicentre study. BMC Anesthesiol..

[15] Popa F., Arafat R., Purcărea V.L., Lală A., Bobirnac G. (2010). Occupational Burnout levels in Emergency Medicine—A nationwide study and analysis. J. Med. Life..

[16] Moscu C.A., Marina V., Anghele M., Anghele A.D., Dragomir L. (2022). Did Personality Type Influence Burn Out Syndrome Manifestations During Covid-19 Pandemic?. Int. J. Gen. Med..

[17] Popa F., Arafat R., Purcărea V.L., Lală A., Popa-Velea O., Bobirnac G. (2010). Occupational burnout levels in emergency medicine—A stage 2 nationwide study and analysis. J. Med. Life..

[18] Bianchi R. (2018). Burnout is more strongly linked to neuroticism than to work-contextualized factors. Psychiatry Resour..

[19] McManus I.C., Keeling A., Paice E. (2004). Stress, burnout and doctors’ attitudes to work are determined by personality and learning style: A twelve year longitudinal study of UK medical graduates. BMC Med..

[20] Swider B.W., Zimmerman R.D. (2010). Born to burnout: A meta-analytic path model of personality, job burnout, and work outcomes. J. Vocat. Behav..

[21] Bakker A., Van der Zee K., Lewig K., Dollard M. (2006). The relationship between the big five personality factors and burnout: A study among volunteer counselors. J. Soc. Psychol..

[22] Alarcon G., Eschleman K.J., Bowling N.A. (2009). Relationships between personality variables and burnout: A meta-analysis. Work. Stress.

[23] Angelini G. (2003). Big five model personality traits and job burnout: A systematic literature review. BMC Psychol..

[24] Kyron M.J., Rees C.S., Lawrence D., Carleton R.N., McEvoy P.M. (2021). Prospective risk and protective factors for psychopathology and wellbeing in civilian emergency services personnel: A systematic review. J. Affect. Disord..

[25] Sterud T., Hem E., Ekeberg O., Lau B. (2008). Occupational stressors and its organizational and individual correlates: A nationwide study of Norwegian ambulance personnel. BMC Emerg. Med..

[26] Mirhaghi A., Mirhaghi M., Oshio A., Sarabian S. (2016). Systematic Review of the Personality Profile of Paramedics: Bringing Evidence into Emergency Medical Personnel Recruitment Policy. Eurasian J. Emerg. Med..

[27] Griffiths M.D., Demetrovics Z., Atroszko P.A. (2018). Ten myths about work addiction. J. Behav. Addict..

[28] Burke R.J., Matthiesen S.B., Pallesen S. (2006). Personality correlates of workaholism. Personal. Individ. Differ..

[29] Kun B., Takacs Z.K., Richman M.J., Griffiths M.D., Demetrovics Z. (2020). Work addiction and personality: A meta-analytic study. J. Behav. Addict..

[30] Merchaoui I., Gana A., Machghoul S., Rassas I., Hayouni M.M., Bouhoula M., Chaari N., Hanchi A., Amri C., Akrout M. (2021). Risk of work addiction in academic physicians prevalence, determinants and impact on quality of life. Int. J. Fam. Community Med..

[31] Rezvani A., Boujou G., Keriven-Dessomme B., Moret L., Grall-Bronnec M. (2014). Workaholism: Are physicians at risk?. Occup. Med..

[32] Puticiu M., Grecu M.-B., Rotaru L.T., Butoi M.A., Vancu G., Corlade-Andrei M., Cimpoesu D., Tat R.M., Golea A. (2024). Exploring Burnout, Work Addiction, and Stress-Related Growth among Prehospital Emergency Personnel. Behav. Sci..

[33] Ruiz-Garcia P., Castanheira A.M., Borges E., Mosteiro-Diaz M.-P. (2022). Workaholism and work-family interaction among emergency and critical care nurses. Intensive Crit. Care Nurs..

[34] Mazzetti G., Chiesa R., Vignoli M., Depolo M., Di Fabio A. (2016). The bad performance of neurotic employees: A matter of job satisfaction and workaholism. Neuroticism: Characteristics, Impact on Job Performance and Health Outcomes.

[35] Jackson S.S., Fung M.-C., Moore M.-A.C., Jackson C.J. (2016). Personality and Workaholism. Personal. Individ. Differ..

[36] Kızıloğlu M., Kircaburun K., Ozsoy E., Griffiths M.D. (2022). Work Addiction and Its Relation with Dark Personality Traits: A Cross-sectional Study with Private Sector Employees. Int. J. Ment. Health Addict..

[37] Spurk D., Hirschi A., Kauffeld S. (2016). A New Perspective on the Etiology of Workaholism: The Role of Personal and Contextual Career-Related Antecedents. J. Career Assess..

[38] Tedeschi R.G., Calhoun L.G. (2004). Posttraumatic Growth: Conceptual Fundations and Empirical Evidence. Psychol. Inq..

[39] Park C.L., Cohen L.H., Murch R.L. (1996). Assessment and Prediction of Stress-Related Growth. J. Personal..

[40] Manole E.C., Curșeu P.L. (2024). Stress-related growth in the early stages of the COVID-19 pandemic: Evidence from a panel study. Personal. Individ. Differ..

[41] Chen R., Sun C., Chen J., Jen H., Kang X.L., Kao C., Chou K. (2021). A Large-Scale Survey on Trauma, Burnout, and Posttraumatic Growth among Nurses during the COVID-19 Pandemic. Int. J. Ment. Health Nurs..

[42] Brooks S., Amlôt R., Rubin G.J., Greenberg N. (2020). Psychological resilience and post-traumatic growth in disaster-exposed organisations: Overview of the literature. BMJ Mil. Health..

[43] Bi X., Proulx J., Aldwin C.M., Friedman H. (2006). Stress-related growth. Encyclopedia of Mental Health.

[44] Park C.L. (1998). Stress-related growth and thriving through coping: The roles of personality and cognitive processes. J. Soc. Issues.

[45] Shakespeare-Finch J., Gow K., Smith S. (2005). Personality, coping and posttraumatic growth in emergency ambulance personnel. Traumatol..

[46] Zuckerman M., Kuhlman D.M., Joireman J., Teta P., Kraft M. (1993). A comparison of the three structural models of personality: The big three, the big five, and the alternative five. J. Personal. Soc. Psychol..

[47] Zuckerman M., de Raad B., Perugini M. (2002). Zuckerman-Kuhlman personality questionnaire (ZKPQ): An alternative five-factorial model. Big Five Assessment.

[48] Barchard K.A. The Levels of Emotional Awareness Scale and Emotional Expressivity. Unpublished Ph.D. Thesis, University of British Columbia, Vancouver, BC. https://img.faculty.unlv.edu/lab/conference-presentations/conference%20posters/LEASandEEWPA2001.pdf.

[49] Iliescu D., Popa M., Dimache R. (2005). Adaptarea romaneasca a Setului International de Itemi de Personalitate: IPIP-Ro. Psihol. Resur. Um..

[50] Demerouti E., Mostert K., Bakker A.B. (2010). Burnout and work engagement: A thorough investigation on the independency of both constructs. J. Occup. Health Psychol..

[51] Schaufeli W.B., Akihito S., Taris T.W. (2009). Being driven to work excessively hard: The evaluation of a two-factor measure of workaholism in the Netherlands and Japan, Cross-Cultural Research. J. Comp. Soc. Sci..

[52] del Libano M., Llorens S., Salanova M., Schaufeli W. (2010). Validity of a brief workaholism scale. Psichotema.

[53] Miclea M., Porumb M., Cotârlea P., Albu M. (2009). Personalitate și interese. CAS++ Cognitrom Assesment System.

[54] Brewer E.W., Shapard L. (2004). Employee Burnout: A Meta-Analysis of the Relationship Between Age or Years of Experience. Hum. Resour. Dev. Rev..

[55] Johnson S.J., Machowski S., Holdsworth L., Kern M., Zapf D. (2017). Age, emotion regulation strategies, burnout, and engagement in the service sector: Advantages of older workers. Rev. Psicol. Trab. Y Organ..

[56] Marchand A., Blanc M.-E., Beauregard N. (2018). Do age and gender contribute to workers’ burnout symptoms?. Occup. Med..

[57] Geuens N., Van Bogaert P., Franck E. (2017). Vulnerability to burnout within the nursing workforce. The role of personality and interpersonal behavior. J. Clin. Nurs..

[58] Masoumi B., Heydari F., Boroumand A.B., Nasr Isfahani M., Izadi Dastgerdi E., Fereidouni Golsefidi A. (2024). Personality factors associated with burnout in the nursing profession during the COVID-19 pandemic. Adv. Biomed. Res..

[59] Armon G., Shirom A., Melamed S. (2012). The big five personality factors as predictors of changes across time in burnout and its facets. J. Personal..

[60] Martin S.R., Fortier M.A., Heyming T.W., Ahn K., Nichols W., Golden C., Saadat H., Kain Z.N. (2022). Perfectionism as a predictor of physician burnout. BMC Health Serv. Res..

